# On five species of the tribes Abacetini and Pterostichini (Coleoptera, Carabidae)

**DOI:** 10.3897/zookeys.352.6294

**Published:** 2013-11-19

**Authors:** Borislav V. Guéorguiev

**Affiliations:** 1 1 National Museum of Natural History, 1 Blvd. Tzar Osvoboditel, 1000 Sofia, Bulgaria

**Keywords:** Coleoptera, Carabidae, Abacetini, Pterostichini, taxonomy, new species, new combinations, new synonym, Italy, Pakistan, China, Indonesia, Papua New Guinea

## Abstract

*Metabacetus willi* **sp. n.** (type locality: Indonesia, Central Java Province, Purworejo Regency, Kaligesing District, cave Seplawan near Donorejo) and *Rhytiferonia beroni* **sp. n.** (type locality: Papua New Guinea, West Sepik Province, Bonforok bil, Tifalmin, 1600 m) are described. Two new combinations: *Poecilus (Ancholeus) campania* (Andrewes, 1937), **comb. n.** of *Feronia campania* Andrewes, 1937, *Aristochroa poecilma* (Andrewes, 1937), **comb. n.** of *Feronia poecilma* Andrewes, 1937, and a new synonymy: *Pterostichus (Oreophilus) podgoricensis* B. Guéorguiev, 2013, **syn. n.** of *Pterostichus (Oreophilus) flavofemoratus pinguis* (Dejean, 1828), are proposed, too.

## Introduction

This paper announces results achieved by the author during the work with the carabid collection of the National Museum of Natural History, Sofia and in a visit in the Natural History Museum, London in 2009. In the first institution, we found out two new species among the materials collected by the former director of the museum Petar Beron in Indonesia and Papua New Guinea. In the second institution, we revised the types of two species described in the genus *Feronia* Latreille, 1816 by Herbert Andrewes (Andrewes 1937). These species were described on female specimens and had never been reviewed. In addition, we announce a synonymy of species described recently by ourselves (Guéorguiev 2013).

## Material and methods

Except for the above mentioned material, a few other species have been studied. They are listed in the text below.

The measurements and part of drawings were made with an ocular micrometer mounted on a stereoscopic binocular microscope Olympus SZ 60. Another part of the drawings were done with a stereoscopic microscope Carl Zeiss Jena Technival 2.

Measurements: body length from the apex of the longer mandible in closed position to the apex of the longer elytron (BL); body width as maximum distance across body (BW); maximum linear distance across the head, including the eyes (HW); length of pronotum, measured along the midline, from the apical margin to the basal margin (PL); maximum width of pronotum (PW); width of the pronotal apex, between the tips of the fore angles (PaW); width of the pronotal base, between the tips of the hind angles (PbW); length of elytra, from a line connecting the apices of the humeral angles to the apex of the longer elytron (EL); maximum width of elytra (EW).

### The examined material is deposited in the following collections

BMNH Natural History Museum, London, United Kingdom (Max Barclay, Beulah Garner)

EMEC Essig Museum of Entomology, University of California, Berkeley, USA (Peter Oboyski)

MCNM Museo Civico di Storia Naturale, Milano (Maurizio Pavesi)

MCSN Museo Civico di Storia Naturale “Giacomo Doria”, Genova, Italy (Maria Tavano)

MNHUB Museum für Naturkunde der Humboldt-Universität, Berlin, Germany (Manfred Uhlig, Bernd Jaeger)

NHRS Swedish Museum of Natural History, Stockholm, Sweden (Johannes Bergsten)

NMNHS National Museum of Natural History, Sofia (Borislav Guéorguiev)

NMW Naturhistorisches Museum Wien, Vienna, Austria (Harald Schillhammer)

The distribution maps were generated using the online mapping software SimpleMappr (©David P. Shorthouse).

## Taxonomic part

### ABACETINI

#### Metabacetus willi sp. n.

http://zoobank.org/0CC6CA65-2C73-4F0D-93E6-42E5A4138BA7

http://species-id.net/wiki/Metabacetus_willi

Figs 1 – 8 , Table 1

##### Type material.

Holotype ♂, “INDONESIA, Java cave Seplawan 2.VI.1994, leg. P. Beron” [typeset], “HOLOTYPE *Metabacetus willi* spec. nov. Guéorguiev des. 2012” [typeset, red label] (NMNHS). Paratypes 4♂♂, 3♀♀, labelled as follow: 3♂♂, 1♀, “INDONESIA, Java cave Seplawan 2.VI.1994, leg. P. Beron” [typeset] (EMEC, BMNH, NMNHS); 1♂, 2♀♀, “INDONESIA, Java v. Kimiri, D.I. Yogyakarta Gua (cave) Nging Rong 29.VIII.1995, P. Beron leg.” [typeset] (BMNH, MCNM, NMNHS); all paratypes with subsequently added: “PARATYPE *Metabacetus willi* spec. nov. Guéorguiev des. 2012” [typeset, red label].

##### Examined type material of other species.

*Metabacetus immarginatus* Bates, 1892, syntype ♀, “Carin Ghecù 1300-1400 m L. Fea II-III.88.” [typeset], “Typus” [red typeset, white label], “*Metabacetus immarginatus* Bates” [handwritten], “*Metabacetus immarginatus* Bates” [handwritten], “*Metabacetus*, n.g. *immarginatus* (es. typ.) Bates” [handwritten, yellow label, genus name underlined], “Syntypus *Metabacetus immarginatus* Bates, 1892” [handwritten & typeset, red label], “Museo Civico di Genova”[typeset] (MCSN). *Mateuellus troglobioticus* Deuve, 1990, 2♂, 1♀, “Indonesia, Sulawesi Selatan, Bantimurung Gua (Cave) Minpiovo 3.IX.1995, P.Beron leg.” (NMNHS).

##### Diagnosis.

**Figure 1.**
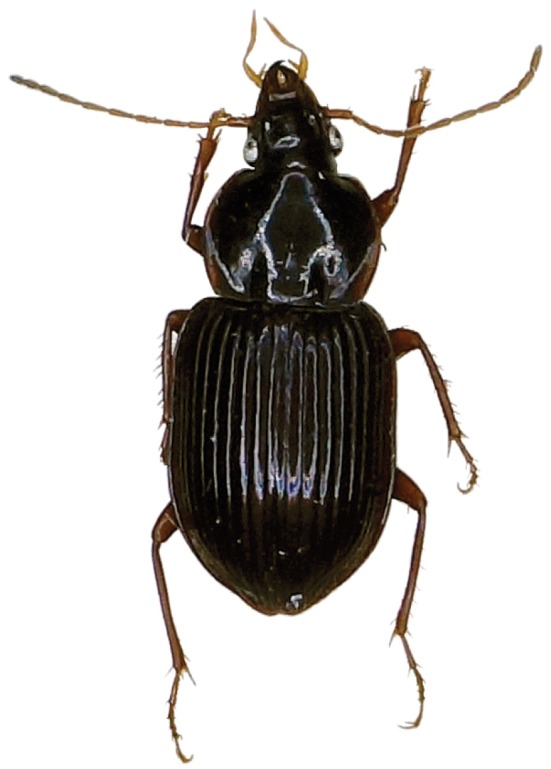
*Metabacetus willi* sp. n., paratype.

**Figures 2–7.**
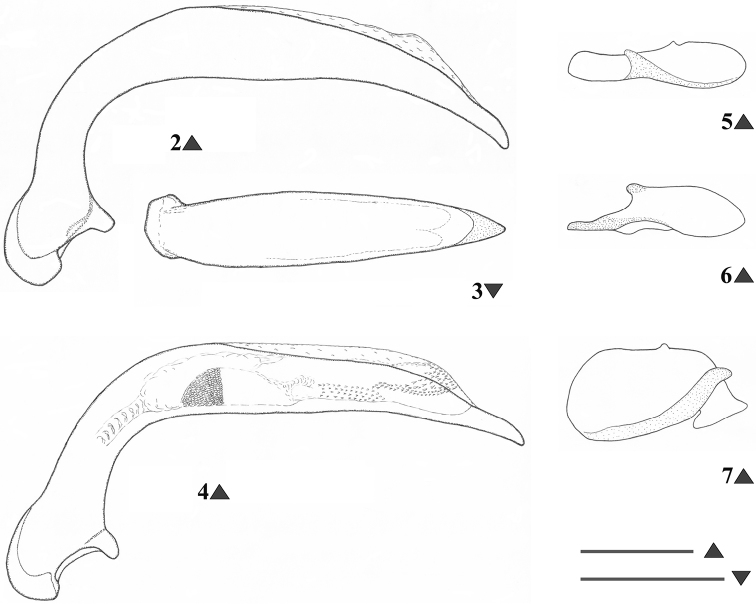
*Metabacetus willi* sp. n., male genitalia (Figs **2–3**, **5–7** holotype, cave Seplawan; Fig. **4** paratype, cave Ngingrong) **2, 4** median lobe of aedeagus, left lateral view **3** median lobe of aedeagus, dorsal view **5** right paramere, internal face **6** right paramere, external face **7** left paramere, internal face. Scale line: 0.3 mm (Figs 2–3; 5–7); 1 mm (Fig. 4).

A medium-sized, slightly iridescent species of *Metabacetus* (Fig. 1), with elongate and attenuate maxillary palpi, last three segments of antennae surpassing the base of pronotum, pronotum widest just after the middle, with anterior margin much shorter than posterior one, sides much narrower anteriorly (than posteriorly), convex posteriorly, lateral fields broadened and moderately reflexed from the middle to the base and obtuse hind angles, prosternum shallowly sulcate medially near apex, apex of elytra without spines, and specific structure of the median lobe of aedeagus (Figs 2–4).

For detailed information about some measurements and ratios see Table 1.

**Table 1.** Data on variation in measurements and ratios among the type series of *Metabacetus willi* sp. n. (mark ‘*’ concerns specimens from cave Ngingrong).

Specimen	BL (mm)	PL (mm)	PW (mm)	EL (mm)	EW (mm)	PW/HW	PW/PL	PW/PbW	PbW/PaW	EW/PW	PL/EL	EL/EW
HT ♂	6.2	1.5	1.9	3.55	2.6	1.71	1.27	1.27	1.32	1.32	0.43	1.39
PT 1♂*	6.2	1.5	1.95	3.5	2.5	1.71	1.3	1.26	1.31	1.29	0.43	1.4
PT 2♂	6.3	1.55	1.95	3.65	2.6	1.75	1.29	1.26	1.34	1.31	0.43	1.39
PT 3♂	6.4	1.5	2.0	3.6	2.6	1.81	1.29	1.26	1.34	1.35	0.43	1.33
PT 4♂	6.1	1.45	1.85	3.55	2.45	1.71	1.31	1.27	1.32	1.30	0.43	1.38
PT 5♀*	6.7	1.5	2.05	3.85	2.8	1.76	1.31	1.24	1.32	1.35	0.42	1.35
PT 6♀*	6.6	1.6	2.1	3.45	2.75	1.77	1.29	1.26	1.33	1.32	0.43	1.37
PT 7♀	6.5	1.6	2.05	3.6	2.6	1.76	1.31	1.28	1.33	1.27	0.43	1.38
**Mean**	**6.375**	**1.53**	**1.98**	**3.59**	**2.61**	**1.748**	**1.296**	**1.263**	**1.326**	**1.314**	**0.429**	**1.374**

##### Description.

*Habitus*. Moderately-sized species of *Metabacetus* Bates, 1892, with sub-oval and convex body. *Measurements*. BL: 6.1–6.7 mm (mean 6.375 mm); BW = EW, see below; PL: 1.45–1.6 mm (mean 1.53 mm); PW: 1.85–2.1 mm (mean 1.98 mm); EL: 3.45–3.85 mm (mean 3.59 mm); EW: 2.45–2.8 mm (mean 2.61 mm). *Ratios*. PW/HW: 1.71–1.81 (mean 1.748); PW/PL: 1.27–1.31 (mean 1.296); PW/PbW: 1.24–1.28 (mean 1.263); PbW/PaW: 1.31–1.34 (mean 1.326); EW/PW: 1.27–1.35 (mean 1.314); PL/EW: 0.42–0.43 (mean 0.429); EL/EW: 1.33–1.4 (mean 1.374). *Color*. Body dark brown to dark reddish dorsally and ventrally, antennae, legs, pronotal margins, and elytral epipleura paler, reddish, palpi yellowish. *Microsculpture and lustre*. Very fine, with transverse microreticulation, distinct on head, pronotum, elytra, and most of ventral surface, visible under magnification > 50×, indistinct on ventral side of head and middle parts of thorax and abdominal sternites; body very shiny throughout, elytra and less ventral surface with slight spectral iridescence. *Head*. Longer than wide, narrow in relation to pronotum; disc smooth, frontal furrows deeply impressed, oblique, divergent backward, not reach level of anterior supraorbital punctures; eyes projecting laterally, temporae as long as half diameter of eyes; two pairs of supraorbital setae; paraorbital sulci moderately deep, surpassing level of posterior supraorbital pore backward; antennae long, filiform, densely pubescent from second fourth of segment 4, with terminal three articles surpassing base of pronotum and apex of last antennomere surpassing anterior fifth of elytron; mandibles elongate, with apex pointed and slightly hooked; labrum rectangular, with six setigerous punctures on anterior margin; clypeus trapezoid, rectilinear anteriorly, with two setigerous punctures closer to lateral margins than to anterior one; glossal sclerite of ligula with two long setae on anterior margin; mentum shallowly emarginated, with simple, widely round at tip tooth, pair of labial setae, and deep labial pits, epilobes short, sub-triangular distally, slightly exceeding mentum tooth forward, mentum separated by submentum by distinct labial suture; submentum with four setae, two basal setae longer than lateral ones; maxillary palpomeres glabrous, elongate and attenuate, larger in comparison with labial palpomeres, as long as two third of head length, apical three segments nearly equal in length; labial palpi fusiform, palpomere 2 longer than palpomeres 3, with two long medial setae. *Pronotum*. Disc-shaped, circular, broader than long, widest just after middle; disc smooth, gently convex medially; midline fine, distinct on medial three fourth of pronotum length, obsolescent apically and basally; anterior sub-marginal sulcus distinct laterally, disappeared medially; anterior and posterior margins of pronotum unbordered, anterior margin scarcely concave, distinctly shorter than posterior one, with fore angles not protruding forward; posterior margin slightly convex backward, hind angles obtuse, incompletely round, not prominent; lateral margins rounded, more anteriorly than posteriorly, without sinuation towards hind angles, lateral fields broadened and moderately reflexed upward towards base, marginal beads continuous, only before hind angles obsolescent; anterolateral seta at anterior second quarter, posterolateral seta at hind angle; posterolateral impressions deep, as long as quarter of pronotum length or so. *Elytra*. Ovoid, wide, rather convex dorsally, slightly narrower basally, with shoulders rounded, widened toward behind as parallel-sided along anterior two thirds, widest along medial third, distinctly sinuate before apex, apices of each elytron rounded at tip; epipleurae with distinct external plicae; striae complete, deeply impressed, internal six striae feebly punctate, striae 7–9 pronouncedly punctate; parascutellar striae present, anastomosing with stria 1; scutellar setigerous pores present, on base of striae 2, slightly removed back from basal margin with distance of diameter of pore or so; basal margin complete; discal setigerous punctures absent; stria 7 with two setigerous punctures near to apex, subapical puncture larger than apical one; intervals moderately convex; umbilicate series of elytra in stria 8, shortly interrupted in middle, consist of 14 setigerous punctures. *Hind wings*. Well-developed. *Ventral surface* (*thorax and abdomen*). Prosternum and proepisterna smooth and glabrous, prosternum only shallowly sulcate medially along apex, prosternal process unbordered; mesosternum smooth, metaepisterna elongate, impunctate, longer than wide, strongly narrowed posteriorly, with wide anterior margins and very short posterior ones; metasternum smooth, only laterally with three-four large punctures from each side, deeply grooved laterally. Abdomen glabrous except one pair paramedial setae on sterna IV–VI, sternum VII with one pair sub-apical setae in males, with two pairs of sub-apical setae in females; sternum II with cluster of deep punctures laterally. *Legs*. Moderately slender and long; protrochanter with one seta; profemur anterior face with a few very short setae, ventral face glabrous, posterior face with three long setae, two medial and one subapical, dorsal face with three-four short setae; mesocoxa with two setae, one medial and one lateral; mesotrochanter with one distal seta; mesofemur anterior face with four long setae, two basal and two medial ones, dorsal face with 7–9 short setae arranged in one-two rows along length, posterior face with several short setae, ventral face glabrous; metacoxa with two lateral setae, one anterior and one posterior, medial transverse sulcus deep and sinuate, not reaching external coxal margin, distant from anterior margin; metatrochanter slightly shorter than half length of metafemur, with one proximal seta, elongate, apex pointed; metafemur anterior face with one basal and one medial (near ventral edge) setae, dorsal face with one rather short seta at distal third, posterior and ventral faces glabrous; structure of pro-, meta-, and metatibia, as well as of tarsomeres in accordance with that described by Will and Park (2008). *Male genitalia* (Figs 2–7). Median lobe of aedeagus with short and bent basal part and long apical part, apex pointed and curved down in lateral view, internal sac with field of numerous sclerotized scales, situated medially and subapically lengthwise (Figs 2–3), blade long, with margins regularly narrowed towards pointed apex in dorsal view (Fig. 4); right paramere elongate, with short triangular, basal process extended inwardly (Figs 5–6), left paramere conchoid, with massive vermiform, basal process inwardly (Fig. 7).

##### Etymology.

A noun in the genitive case. Honours Kippling Will, a notable American carabidologist, for his studies on the Pterostichitae carabids.

##### Distribution.

**Figure 8.**
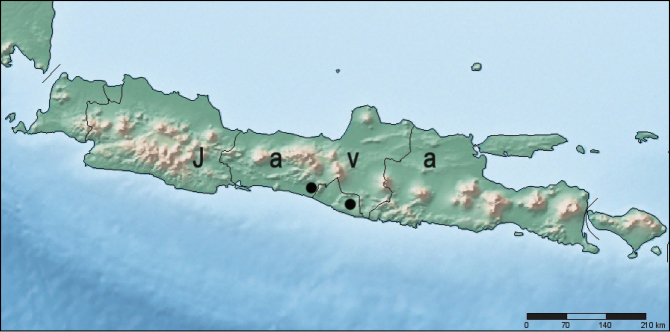
Localities of *Metabacetus willi* sp. n.

So far, this species was only found in two caves in the southern part of Java Island, Indonesia (Fig. 8): cave Seplawan near village Donorejo (Central Java Province, Purworejo Regency, Kaligesing District); cave Ngingrong near village Mulo (Yogiakarta Special Administrative Region, Gunung Kidul Regency).

##### Affinities.

Due to the present knowledge of the taxonomy of the genus and its related taxa, it is difficult to identify the adelphotaxon of the new taxon or to state the most related taxa to it, moreover some unnamed species (see Will and Park 2008: 189–190, Fig. 1) await adequate examination. Based on selected features, namely moderately large size of body, slightly iridescent tegument, sides of pronotum convex posteriorly, with lateral fields broadened and moderately reflexed towards the base, prosternum shallowly sulcate medially near apex, apex of elytra without evident spines, *Metabacetus willi* sp. n. seems closer to *Metabacetus laotinus* Straneo, 1938 (Laos) and *Metabacetus immarginatus* s.l. (India: West Bengal and North Burma) than to the other congeners. However, the species from South Java well differs from the last two species in the presence of more elongate appendages (especially long maxillary palpi), segments IX-XI of antennae reaching beyond the base of pronotum, pronotum widest after the middle, with anterior margin much shorter than posterior one, sides much narrower anteriorly than posteriorly, and obtuse hind angles.

##### Ecological remarks.

*Metabacetus willi* sp. n. is the first member of the genus which can be classified as trogloxene (or troglophile). Although slight, it shows several morphological adaptations to cave-dwelling. Compared with the other congeners, its eyes are less protruding, with ommatidia less numerous, its appendages (namely, the maxillary palpi, antennomeres, and legs) a bit longer, and body less robust in sagittal plan and more flattened along the dorsoventral axis. However, the flight wings of the new species are still well-developed and it seems capable of flight. Most probably, this beetle lives not only in caves, but also on the forest floor of woodlands outside the cave systems.

**Figure 9.**
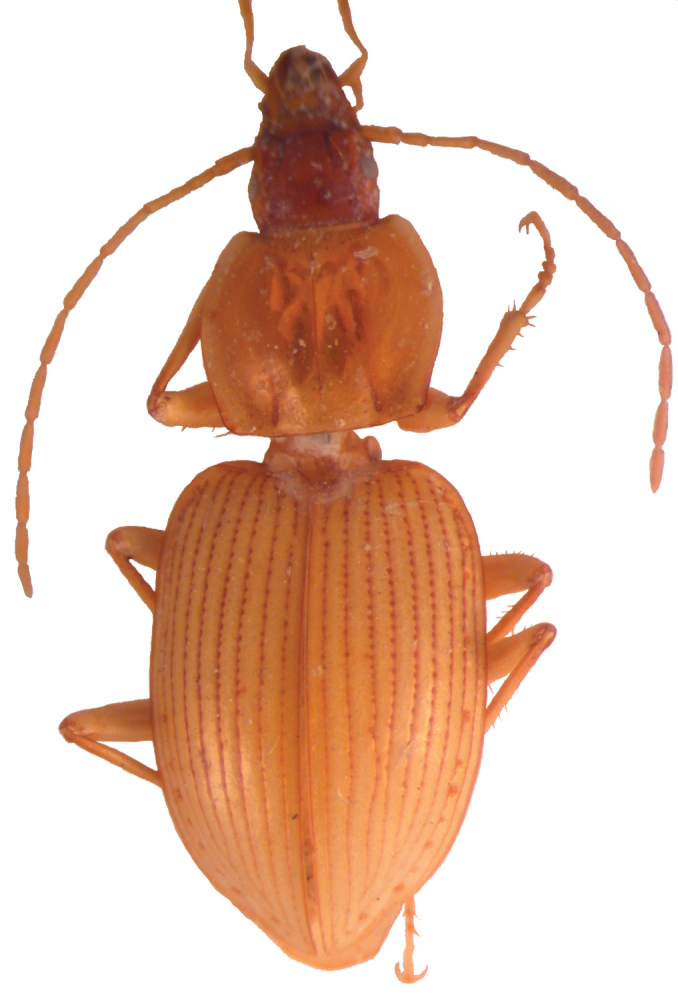
*Mateuellus troglobioticus* Deuve, holotype.

Another related species, *Mateuellus troglobioticus* (Fig. 9), which together with *Metabacetus* belongs to the same clade (Will 2006, Will and Park 2008: 190), displays predominant trilobite mode of life. For the time being, this form has been found three times in caves only (Deuve 1990, present paper).

### PTEROSTICHINI

#### Poecilus (Ancholeus) campania

(Andrewes, 1937) comb. n.

http://species-id.net/wiki/Poecilus_campania

Feronia campania Andrewes, 1937: 3 (type locality: “Punjab: Lyallpur…; Lahore, Shahdara…. India”)

##### Type material.

Holotype ♀, “Type” [typeset, round white label with red margin], “Light Collection Lyallpur. 3.IX.29” [typeset], “*Feronia campania* Andr. Type H. E. Andrewes det.” [handwritten & typeset] (BMNH, box No 706). Paratype ♀, “India” [typeset], “1765” [typeset], “Bowring. 63. 47*” [typeset], “Ex. coll. Brit. Mus.” [typeset], “Co-type” [typeset, round white label with green margin], “*Feronia campania* Andr. cotype H. E. Andrewes det.” [handwritten & typeset], “H. E. Andrewes Coll. B.M.1945-97.” [typeset] (BMNH, box No 706).

##### Other material.

1♀, “Punjab Lyallpur 30.VI.1929 Govt. Entom. Light collection”, “*Feronia campania* Andr. H. E. Andrewes det.” (BMNH, box No 706).

##### Remarks.

The study of the three females showed that they are conspecific and belong to the genus *Poecilus* Bonelli, 1810. According to several character states: 1/ antennal segment 3 compressed and carinate on internal margin; 2/ onychium without ventral setae; 3/ abdominal sternites 4-6 without distinct transverse furrows along base; 4/ pronotum with two basal impressions at each side, the taxon is best placed in the subgenus *Ancholeus* Dejean, 1828. By the structure of the body, color, and corporal dimensions, *Poecilus campania* seems rather similar to *Poecilus wollastoni* (Wollaston, 1854). However, without a thorough revision of the taxa from *Ancholeus* any opinion for eventual relationship between these species will be tentative. For the time being, the *Poecilus campania* is known only from Pakistan (Faisalabad; Lahore).

#### Rhytiferonia beroni sp. n.

http://zoobank.org/9DAC8D91-E7AE-4C20-BACB-A703133F19C7

http://species-id.net/wiki/Rhytiferonia_beroni

Figs 10 – 14

##### Type material.

Holotype ♀, “New Guinea, Bonforok bil, Tifalmin, 1600 m 17.10.75 leg. P. Beron” [typeset], “HOLOTYPE *Rhytiferonia beroni* spec. nov. Guéorguiev des. 2012” [typeset, red label] (NMNHS). Paratypes 2♀♀, labeled as follows: 1♀, “Tifalmin W. Sepic Prov. IX.75” [handwritten], “British Speleological Expedition to Papua New Guinea 1975” [typeset], “*Rhytiferonia* sp. det. B. P. Moore ’78” [handwritten & typeset] (NMNHS); 1♀, “Fimin tel 2300 m Western Prov. 30.viii.75 P. Beron” [handwritten], “British Speleological Expedition to Papua New Guinea 1975” [typeset], “*Rhytiferonia* sp. det. B. P. Moore ’78” [handwritten & typeset], “UC Berkeley EMEC 345536” [typeset]; paratypes with subsequently added: “PARATYPE *Rhytiferonia beroni* spec. nov. Guéorguiev des. 2012” [typeset, red label] (EMEC).

##### Examined type material of other species.

*Rhytiferonia julianae* Baehr, 2001, paratypes 2♂♂, “IRIAN JAYA: Mt. Juliana Gebiet 16.–17.9.1993 Sab-me Tal” [typeset], “ca. 140°17'E, 04°27'S, 3400–3500m leg. M. Balke (14)” [typeset], “PARATYPE Rhytiferonia julianae, sp.nov. det. M. Baehr 2000” [typeset, red label] (NMW).

##### Diagnosis.

The new species is distinct from all other congeners in the following set of characters: 1) eyes moderately enclosed by temporae laterally; 2) pronotum with obtuse, somewhat perceptible basal angles, side without sinuation in front of angle; 3) posterolateral seta of pronotum slightly removed from angle; 4) elytra with parascutellar setigerous puncture, distinct parascutellar stria in interval 1, and angular base of stria 1 joining stria 2; 5) last three abdominal sterna with transverse sulci superficial, only laterally distinct.

It should be noted that the above diagnosis is based on the descriptions of the known species by Baehr (2001) as specimens of only *Rhytiferonia* species were used for comparison (see chapter “Examined type material of other species”).

##### Description.

**Figure 10.**
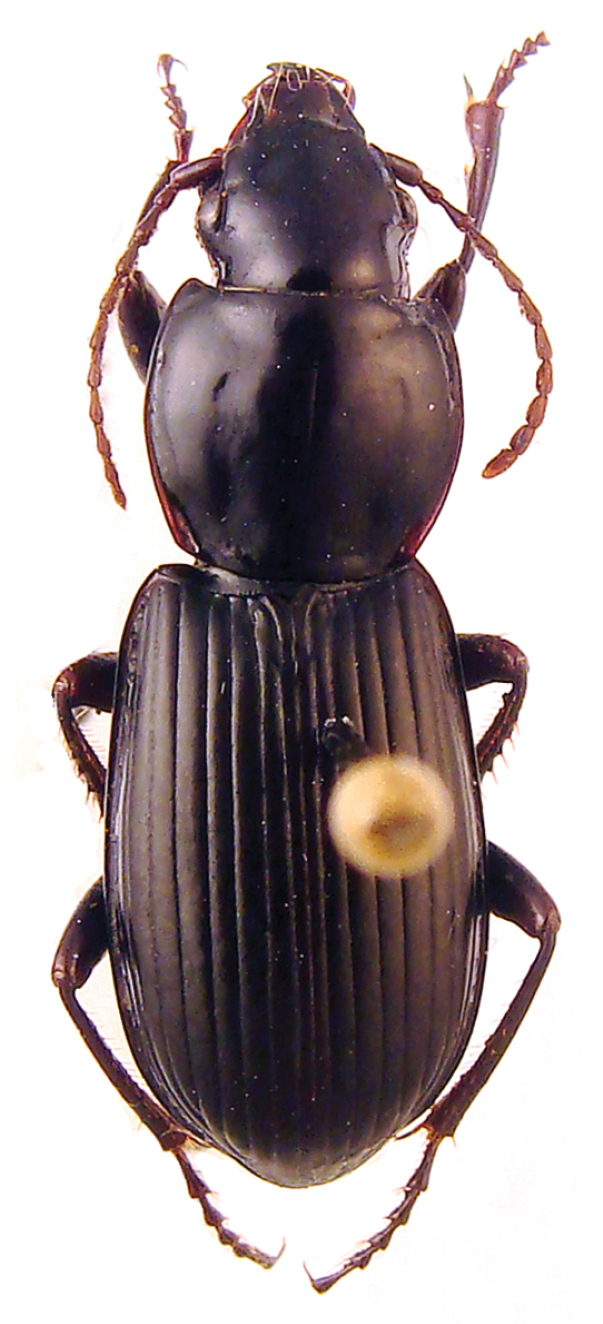
*Rhytiferonia beroni* sp. n., holotype.

**Figures 11–13.**
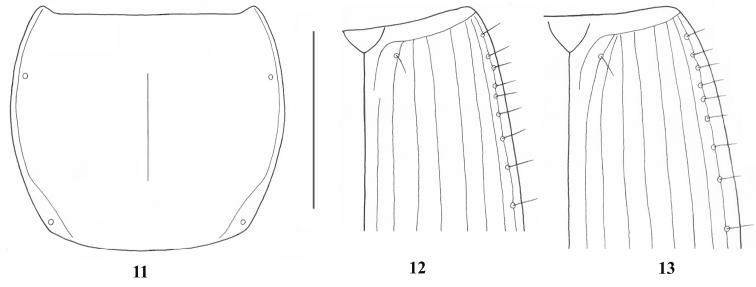
*Rhytiferonia beroni* sp. n. **11** pronotum, holotype **12–13** base of elytra (**12** holotype **13** paratype). Scale line: 3 mm.

*Habitus*. Moderately large-sized species of *Rhytiferonia*, with elongate, convex body and basal angles of pronotum rounded off (Fig. 10). *Measurements*. BL: 15.6–17.2 mm (17.1 mm in holotype); BW: 4.9–5.5 mm (5.5 mm in holotype). *Ratios*. PW/HW: 1.38–1.42 (1.42 in holotype); PW/PL: 1.10–1.16 (1.16 in holotype); PW/PbW: 1.35–1.37 (1.37 in holotype); PbW/PaW: 0.98–1.00 (0.98 in holotype); EW/PW: 1.20–1.23 (1.20 in holotype); EL/EW: 1.62–1.68 (1.62 in holotype). *Color*. Deep black on dorsal surface, mouthparts, antennae, legs, and ventral surface black brown to dark reddish. *Microsculpture and lustre*. Very fine, isodiametric, distinct on head and elytra, visible under magnification > 50 x, indistinct on pronotum; dorsal surface shiny. *Head*. Longer than wide, disc smooth, frontal furrows faintly impressed, oblique, divergent backward, hardly reach level of anterior supraorbital punctures; eyes small, modestly projecting laterally, as long as temporae; temporae slightly surpassing eyes laterally; paraorbital sulci moderately deep, reaching level of posterior supraorbital pore backward; labrum rectangular, anterior margin with six setigerous punctures; clypeus trapezoid, slightly emarginated anteriorly and laterally, with two setigerous punctures closer to lateral margins than to anterior one, clypeal suture faint; antennae filiform, pubescent from second fifth of segment 4, with terminal article not reaching base of pronotum; glossal sclerite of ligula with two long setae on anterior margin; maxillary palpomere 1 very massive, twice thicker than following two segments; mentum deeply emarginated, with tooth bifid at tip and pair of labial setae, epilobes large, significantly exceeding mentum tooth forward; submentum with two basal setae, without lateral ones. *Pronotum* (Fig. 11). Large, widest at middle, disc gently convex, smooth; midline very fine, distinct on medial half of pronotum, obsolescent apically and basally; anterior and posterior margins unbordered, almost of equal length, anterior margin slightly concave, fore angles moderately protruding forward; basal margin rather convex backward, hind angles subangular, incompletely round; lateral margins slightly convex to straight, without sinuation towards hind angles, marginal field narrow in apical half, widened and explanate towards base; anterolateral seta at apical third, posterolateral seta slightly in front of hind angles; posterolateral impressions faint. *Elytra* (Figs 12–13). Subelongate, oviform, convex dorsally, coalescent along suture, widest at third fourth; sides narrow basally, gradually widened apically; shoulders angulate, with minute teeth; lateral margins slightly sinuate before rounded apex; striae well impressed, complete, impunctate, parascutellar striae present, not anastomosing with stria 1 back, angular base of stria 1 present, joining stria 2 (in paratype) or reduced (in holotype), scutellar pores present, removed back from basal margin with distance from one to three diameters of pore, situated on angular base of stria 1, stria 7 deepened in apical third, with one setigerous puncture near to apex, discal setigerous punctures absent; intervals gently convex; umbilicate series of elytra entire, not interrupted in middle, consist of 19–21 setigerous punctures. *Hind wings*. Vestigial. *Ventral surface* (*thorax and abdomen*). Prosternum laterally with conspicuous longitudinal sulci; prosternal process unbordered; metaepisterna wider than long, with anterior margins longer than inner ones and as long as outer margins; apical three abdominal sternites with transverse sulci superficial, distinct laterally, indistinct in middle; last visible sternite quadrisetose in female. *Legs*. Fore and middle legs relatively short and massive, hind legs longer; fore and middle trochanteri with one seta, hind trochanteri asetose, with pointed apex, as long as half of hind femora; fore coxae asetose, middle coxae with two setae, hind coxae with three setae, including medial setae after meeting point of coxae; profemur posterior margin with four pores, mesofemur posterior margin with five pores, metafemur anterior margin with three pores; onychium setose ventrally. *Female genitalia*. Not studied.

##### Etymology.

A noun in the genitive case. Honour Dr. Petar Beron, a prominent Bulgarian zoologist, who first collected the new species.

##### Distribution.

**Figure 14.**
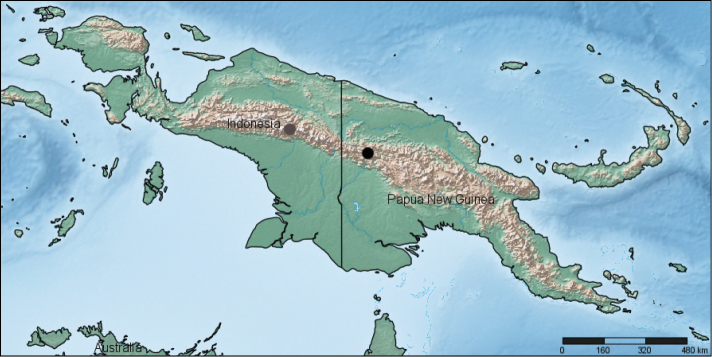
Localities of *Rhytiferonia punctigera* (grey circle) and *Rhytiferonia beroni* sp. n. (black circle).

Papua New Guinea, Sandaun Province (= West Sepik Province), Telefomin District, Tifalmin env. (Fig. 14). For the time being, it is the first documented representative of *Rhytiferonia* Darlington, 1962 from Papua New Guinea.

##### Affinities.

The new species is provisionally placed in the *nigra*-group, which includes *Rhytiferonia nigra* Darlington, 1962, *Rhytiferonia iebele* Darlington, 1962, and *Rhytiferonia punctigera* Baehr, 2001. Baehr (2001: 43) associated the species from this complex due to the presence of: 1/ markedly enclosed eyes; 2/ basal angles of pronotum rounded off, without sinuation in front of them; 3/ posterolateral seta of pronotum far removed from angle; 4/ complete, deep, sharply impressed transverse sulci on three apical abdominal sterna; 5/ median lobe of aedeagus narrow, elongate, little curved medially, without spiniform sclerites near apex of internal sac. As the male genital characters of the new species are unknown, it possesses only two from the remaining four features: markedly enclosed eyes (i) and basal angles of pronotum rounded off, without sinuation in front of angles (ii). In contrast to that, *Rhytiferonia beroni* sp. n. possesses posterolateral seta of pronotum only slightly removed from basal angle and apical three abdominal sternites with superficial, distinct only laterally transverse sulci.

Except for the characters shared by the new species with the species from the *nigra* group, *Rhytiferonia beroni* sp. n. most resembles *Rhytiferonia punctigera* in: 1) the pronotum with obtuse basal angles; 2) presence of parascutellar pore; 3) presence of parascutellar stria, which not anastomose with stria 1, and angular base of stria 1 joining stria 2. This set of shared traits places the last two species closer to each other than any of them to another species of *Rhytiferonia*. The new species can be distinguished from its closest congener in the following row:

1) temporae (“orbits”, after Baehr 2001) laterally slightly surpassing eyes, vs. laterally perceptibly surpassing eyes in *Rhytiferonia punctigera* (Baehr 2001: 45, 55, fig. 16);
2) pronotum widest in the middle, with fore angles moderately protruding (Fig. 11), vs. widest in anterior third, with fore angles little produced in *Rhytiferonia punctigera* (Baehr 2001: 45, 54, fig. 9);
3) anterior and posterior margins of pronotum almost of equal length, PbW/PaW: 0.98, vs. “Base clearly narrower than apex.” (Baehr 2001: 45) in *Rhytiferonia punctigera*;
4) posterolateral seta of pronotum slightly removed from hind angle (Fig. 11), vs. far removed from hind angle in *Rhytiferonia punctigera* (Baehr 2001: 45, 54, fig. 9);
5) transverse sulci on three apical abdominal sternites superficially impressed laterally, indistinct in middle, vs. complete and deep, sharply impressed in *Rhytiferonia punctigera* (Baehr 2001: 45);
6) umbilicate series of 19–21 setigerous punctures, vs. umbilicate series of 18 setigerous punctures in *Rhytiferonia punctigera* (Baehr 2001: 45).

In addition, several ratios with different values in the two species (*Rhytiferonia punctigera* in brackets): PW/HW: 1.38–1.42 (vs. 1.27); PW/PL: 1.10–1.16 (vs. 1.07); PW/PbW: 1.35–1.37 (vs. data questionable, 1.24, according to Baehr 2001: 46, but 1.32, according to Baehr 2001: 52); EL/EW: 1.62–1.68 (vs. 1.79).

#### Key to the species of *nigra*-group

**Table:** 

1	Elytra with parascutellar pore; parascutellar stria present, not anastomosing with stria 1; angular base of stria 1 present, joining stria 2 forward or reduced (Baehr 2001: 55, fig. 20; present work, Figs 12–13)	2
–	Elytra without parascutellar pore; parascutellar stria present, anastomosing with stria 1; angular base of stria 1 absent (Baehr 2001: 55, fig. 19)	3
2	Basal angles of pronotum rounded off at tip; abdominal sternites 4–6 with transverse sulci complete and deep, sharply impressed	*Rhytiferonia punctigera* Baehr, 2001
–	Basal angles of pronotum subangular at tip (Fig. 11); abdominal sterna 4–6 with transverse sulci superficial laterally, indistinct in middle	*Rhytiferonia beroni* sp. n.
3	Pronotum base distinctly narrow than apex, widest diameter in front of middle	*Rhytiferonia nigra* Darlington, 1962
–	Pronotum base almost as wide as apex, widest diameter in middle	*Rhytiferonia iebele* Darlington, 1962

#### Pterostichus (Oreophilus) flavofemoratus pinguis

(Dejean, 1828)

http://species-id.net/wiki/Pterostichus_flavofemoratus_pinguis

Pterostichus (Oreophilus) podgoricensis B. Guéorguiev, 2013: 59 (type locality: “Titograd Yugoslavia”), syn. n.

##### Type material.

Holotype ♂, “NHRS-JLKB 000020046” [typeset], “Titograd Yugoslavia” [handwritten], “coll. J. Ferrer” [handwritten], “HOLOTYPE Pterostichus podgoricensis sp. n. Guéorguiev des. 2012” [typeset red label] (NHRS). Paratype ♀, “Teneriffa Coll. O. Thieme” [typeset], “PARATYPE Pterostichus podgoricensis sp. n. Guéorguiev des. 2012” [typeset red label] (MNHUB). For additional data about these specimens see also Guéorguiev (2013: 59).

##### Other examined material.

*Pterostichus flavofemoratus pinguis*, 4♂♂, 5♀♀, Italy, valley de Gressoney (AO), Fontainemore, 1500 m, forest, 10.VIII.2004, leg. & det. G. Allegro (NMNHS).

##### Remarks.

*Pterostichus podgoricensis* has been described by the author after two specimens (Guéorguiev 2013). Then I regarded this taxon as most allied to *Pterostichus flavofemoratus* (Dejean, 1828) and *Pterostichus spinolae* (Dejean, 1828). Following the publication, two colleagues (see Acknowledgements) informed me that my species may be conspecific with *Pterostichus flavofemoratus pinguis*, a form that I uncritically considered synonymous with *Pterostichus flavofemoratus*. The study of material of *Pterostichus flavofemoratus pinguis* from the Pennine Alps sent me by Gianni Allegro and its comparison with *Pterostichus podgoricensis* showed that the two taxa are identical.

#### Aristochroa poecilma

(Andrewes, 1937) comb. n.

http://species-id.net/wiki/Aristochroa_poecilma

Feronia poecilma Andrewes, 1937: 5 (type locality: “S.E. Tibet: Tsangpo Valley, Nyima La, 15,000 feet”)

##### Type material.

Holotype ♀, “Type” [typeset, white round label with red margin], “Brit. Mus. 1925-189.” [typeset], “S.E.Tibet: Tsangpo Valley, Nyima La. 15,000 22.VI.1924. F. Kingdon Ward.” [handwritten], “*Feroniapoecilma* Andr. Type H. E. Andrewes det.” [handwritten & typeset] (BMNH, box No 682).

##### Examined material of other species.

*Aristochroa gratiosa* Tchitcherine, 1898, 1♂, with two labels in Chinese and a third one “Qinhai province China” (BMNH, box No 682); *Aristochroa* sp., 2♀♀, (BMNH, box No 682). The last two specimens with label “E. Tibet: Pochö. 12-16,000 ft. 18-20.vii.1936”, and one of them with second label “*Feroniapoecilma* Andr. Type H. E. Andrewes det.”.

##### Remarks.

The study of the Andrewes’s type has proved that it belongs to the subtribe Trigonognathina Tschitschérine, 1898, which includes *Aristochroa* Tschitschérine, 1898, *Myas* Sturm, 1826 (incl. *Trigonognatha* Motschulsky, 1858), *Steropanus* Fairmaire, 1888, and *Xenion* Tschitschérine, 1902. The presence of the following set of characters: 1/ anterior margin of ligula with four or more setae; 2/ elytra with intervals 1, 3, 5, and 7 wider, more or less distinctly raised and differently colored than the other intervals; 3/ terminal segment of both the maxillary and labial palpi not enlarged, revealed that the only specimen of this taxon belongs to *Aristochroa*.

The holotype of *Aristochroa poecilma* was found at Nyima La, a high-mountain passage in the Nyingchi Prefecture, the southeastern part of the Tibet Autonomous Region, China.

## Supplementary Material

XML Treatment for Metabacetus willi

XML Treatment for Poecilus (Ancholeus) campania

XML Treatment for Rhytiferonia beroni

XML Treatment for Pterostichus (Oreophilus) flavofemoratus pinguis

XML Treatment for Aristochroa poecilma
